# LAG-3 in Diffuse Large B-Cell Lymphoma: Immunohistochemical Expression Patterns and Clinicopathological Correlations

**DOI:** 10.3390/jcm15176888

**Published:** 2026-09-05

**Authors:** Mehmet Doğan, Emine Begüm Coşkun, Mehmet Bakırtaş, Ali Coşkun, Olcay Kandemir

**Affiliations:** 1 Department of Pathology, Ankara Bilkent City Hospital, Ankara Yıldırım Beyazıt University, Ankara 06800, Türkiye; 2 Department of Diagnostic and Molecular Pathology, Queen’s University, Kingston, ON K7L 3N6, Canada; ozyurekeminebegum@gmail.com (E.B.C.); dr_alicoskun@hotmail.com (A.C.); 3 Department of Hematology, Tekirdağ İsmail Fehmi Cumalıoğlu City Hospital, Tekirdağ 59030, Türkiye; drbakirtas@hotmail.com; 4 Department of Pathology, Etlik City Hospital, Ankara 06010, Türkiye; olcaykandemir@yahoo.com.tr

**Keywords:** diffuse large B-cell lymphoma, DLBCL, LAG-3, immune checkpoint, tumor-infiltrating lymphocytes, tumor microenvironment, immunohistochemistry

## Abstract

**Background/Objectives:** Lymphocyte activation gene-3 (LAG-3) is an inhibitory immune checkpoint molecule that may contribute to immune escape in diffuse large B-cell lymphoma (DLBCL). This study aimed to characterize LAG-3 expression in neoplastic cells and tumor-infiltrating lymphocytes (TILs) and to investigate its associations with clinicopathological features and clinical outcome. **Methods:** This retrospective, single-center study included 210 patients with DLBCL diagnosed between 2007 and 2019. LAG-3 expression was evaluated immunohistochemically in whole-tissue sections. Tumor-cell expression was assessed according to the percentage and intensity of positive cells, while LAG-3-positive TILs were quantified as the mean number of positive lymphocytes per high-power field (HPF). Associations with clinicopathological parameters, treatment response, overall survival, and progression-free survival (PFS) were analyzed. **Results:** LAG-3 staining of varying intensity was observed in neoplastic cells in 177 cases (84.3%), and 82 cases (39.0%) were classified as LAG-3 positive using a 10% tumor-cell cut-off. The median number of LAG-3-positive TILs was 5/HPF. Male patients showed a higher percentage of LAG-3-positive tumor cells than female patients (*p* = 0.039). Moderate-to-strong tumor-cell staining was significantly more frequent in cases with >5 LAG-3-positive TILs/HPF than in those with ≤5 TILs/HPF (*p* = 0.018). LAG-3 expression parameters were not significantly associated with treatment response or overall survival. Univariate Cox regression analysis also showed no significant association between overall survival and LAG-3-positive TIL count, tumor-cell LAG-3 percentage, or staining intensity. Exploratory PFS analysis in 66 evaluable patients also showed no significant association with LAG-3 expression parameters. **Conclusions:** LAG-3 expression in DLBCL occurs in both neoplastic cells and TILs. The association between increased LAG-3-positive TILs and stronger tumor-cell expression supports further investigation of LAG-3 across both cellular compartments. No significant association with overall survival was observed in this cohort; however, the limited number of survival events precludes definitive conclusions regarding its prognostic significance. Further studies using standardized assessment methods and larger, clinically well-characterized cohorts are warranted.

## 1. Introduction

Diffuse large B-cell lymphoma (DLBCL) is the most common subtype of non-Hodgkin lymphoma and is characterized by considerable biological and clinical heterogeneity [1]. Although rituximab-based immunochemotherapy, particularly R-CHOP (rituximab, cyclophosphamide, doxorubicin, vincristine, and prednisone), has substantially improved outcomes, a considerable proportion of patients develop refractory or relapsed disease [1]. The International Prognostic Index (IPI) remains widely used for clinical risk stratification; however, the heterogeneous outcomes observed among patients with DLBCL have encouraged the search for additional biological and prognostic biomarkers [1,2].

The tumor microenvironment (TME) plays an important role in the biology of DLBCL and comprises a complex network of immune and stromal cells that interact with neoplastic B cells. These interactions can influence tumor-cell survival and proliferation, immune evasion, treatment response, and clinical outcome [3,4,5]. Among the mechanisms contributing to immune escape in DLBCL, inhibitory immune checkpoint pathways are particularly relevant because they can suppress effective antitumor T-cell responses and may contribute to the persistence of an immunosuppressive tumor microenvironment while also representing potential therapeutic targets [4].

Lymphocyte activation gene-3 (LAG-3; CD223) is an inhibitory immune checkpoint receptor expressed predominantly on activated and exhausted T cells and other immune-cell subsets. LAG-3 interacts with major histocompatibility complex class II and other ligands and contributes to the negative regulation of T-cell activation and function [6]. Its upregulation in the tumor microenvironment has therefore attracted considerable interest as a mechanism of immune escape and as a potential target for cancer immunotherapy [7,8].

In DLBCL, LAG-3 expression has been demonstrated in tumor-infiltrating lymphocytes (TILs), while expression in neoplastic B cells has also been reported [9,10]. However, the reported extent and distribution of LAG-3 expression vary among studies, and its relationship with clinicopathological characteristics and clinical outcome remains incompletely defined [9,10].

Previous studies have demonstrated heterogeneous patterns of LAG-3 expression in DLBCL. Chen et al. reported frequent LAG-3 expression in TILs but only limited expression in neoplastic cells, whereas Keane et al. demonstrated LAG-3 expression across multiple immune-cell subsets as well as in a proportion of malignant B cells and reported an association between high LAG-3 expression and inferior outcome [9,10]. More recently, Ma et al. reported an association between high LAG-3 expression in TILs and poorer survival [11]. These differences in cellular distribution, assessment methodology, and reported prognostic associations highlight the need for integrated studies evaluating LAG-3 in both neoplastic and immune-cell compartments within the same pathological cohort. Such an approach may help clarify the relationship between LAG-3 expression in these two compartments and its clinical significance.

In this study, we evaluated LAG-3 expression by immunohistochemistry in both neoplastic cells and TILs in a cohort of 210 patients with DLBCL. We investigated the associations between LAG-3 expression patterns and clinicopathological parameters, treatment response, and overall survival to assess its potential clinical and prognostic significance.

## 2. Materials and Methods

This retrospective study included patients diagnosed with DLBCL between 2007 and 2019. Cases from nodal and extranodal sites were identified from the pathology archives of Dr. Abdurrahman Yurtaslan Ankara Oncology Training and Research Hospital. Pathology reports, formalin-fixed paraffin-embedded (FFPE) tissue blocks, and hematoxylin and eosin (H&E)-stained sections were retrieved from the pathology archive. Demographic and follow-up data were obtained from the hospital patient management system, and available clinical data, including treatment response and vital status, were obtained from the Hematology Department. Available clinical data included Ann Arbor stage, International Prognostic Index (IPI), serum lactate dehydrogenase (LDH), Eastern Cooperative Oncology Group (ECOG) performance status, extranodal and bone marrow involvement, B symptoms, treatment regimen, treatment response, and cell-of-origin classification. Because of the retrospective nature of the study, these variables were not available for all patients and were analyzed using the available-case approach. The number of evaluable patients for each variable is reported in the Section 3 and Table 1.

Cases diagnosed with DLBCL between 2007 and 2019 were identified from the pathology archives. DLBCL diagnoses were reviewed and classified according to the 2017 World Health Organization (WHO) classification of lymphoid neoplasms [12]. Following histopathological review, cases were eligible if the diagnosis of DLBCL could be confirmed and adequate, well-preserved FFPE tumor tissue was available for immunohistochemical evaluation. Cases with insufficient or poorly preserved tumor tissue or in which the diagnosis could not be confirmed on review were excluded. A total of 210 cases fulfilled these criteria and were included in the study. When more than one paraffin block was available, a representative block containing an adequate amount of viable tumor tissue was selected.

The study was approved by the Ethics Committee of Dr. Abdurrahman Yurtaslan Ankara Oncology Training and Research Hospital (approval number: 2019-02/201).

### 2.1. Immunohistochemistry and Evaluation of LAG-3 Expression

Whole-tissue sections, 2–4 μm in thickness, were obtained from the selected FFPE blocks and mounted on positively charged slides. Immunohistochemical staining was performed using a recombinant rabbit monoclonal anti-LAG-3 antibody [EPR4392(2), C-terminal; Abcam (Cambridge, UK)] at a dilution of 1:1000 on a Ventana BenchMark XT automated staining platform using the UltraView Universal DAB Detection Kit (Roche Diagnostics, Rotkreuz, Switzerland). Human tonsil tissue was used as a positive control.

All LAG-3-stained slides were jointly evaluated by two pathologists, and final assessments were reached by consensus. The percentage of LAG-3-positive neoplastic cells was estimated by evaluating the whole tumor area. Tumor-cell staining intensity was categorized as absent (0), weak (1+), moderate (2+), or strong (3+).

For assessment of LAG-3-positive TILs, the entire slide was initially screened, and LAG-3-positive lymphocytes were counted in 3–4 high-power fields (HPFs) showing the highest density of positive TILs. The mean number of LAG-3-positive TILs per HPF was then calculated.

Tumor-cell LAG-3 positivity was defined as LAG-3 expression in ≥10% of neoplastic cells, consistent with the threshold used by Chen et al. [10].

For exploratory categorical analyses, cohort-derived median values were used to stratify cases according to tumor-cell LAG-3 percentage (≤6% vs. >6%) and LAG-3-positive TIL count (≤5 vs. >5 TILs/HPF). These median-based categorizations were considered exploratory, and analyses using the original continuous or ordinal LAG-3 variables were also performed, as appropriate.

### 2.2. Statistical Analysis

Statistical analyses were performed using SPSS Statistics version 17.0 (IBM Corp., Armonk, NY, USA). Distribution of quantitative variables was assessed using the Kolmogorov–Smirnov test. Descriptive statistics were expressed as mean ± standard deviation or median and range, as appropriate, whereas categorical variables were summarized as frequencies and percentages.

Variables not meeting parametric assumptions were analyzed using non-parametric methods. The Mann–Whitney U test was used to compare continuous or ordinal variables between independent groups. Associations between categorical variables were assessed using Pearson’s chi-square test, continuity-corrected chi-square test, or Fisher’s exact test, as appropriate according to expected cell counts. Correlations between LAG-3 parameters and continuous or ordinal clinicopathological variables were assessed using Spearman’s rank correlation coefficient.

Overall survival was analyzed using the Kaplan–Meier method, and survival distributions between groups were compared using the log-rank test. The effects of LAG-3-positive TIL count, tumor-cell LAG-3 percentage, and tumor-cell staining intensity on overall survival were further evaluated using univariate Cox proportional hazards regression analysis, with hazard ratios (HRs) and 95% confidence intervals (CIs) reported. Progression-free survival (PFS) was evaluated in patients with available follow-up data. The proportional hazards assumption was assessed using time-dependent covariate interactions and was not violated for the evaluated LAG-3 variables. Progression or death was considered an event, and patients without an event were censored at the last follow-up.

A two-sided *p* value < 0.05 was considered statistically significant.

## 3. Results

A total of 210 DLBCL cases were included, comprising 102 male (48.6%) and 108 female (51.4%) patients. Treatment-response data were available for 69 patients: 48 (69.6%) achieved complete response, 3 (4.3%) achieved partial response, and 18 (26.1%) showed no response. At the end of follow-up, 185 patients (88.1%) were alive, and 25 (11.9%) had died. Complete follow-up time data required for overall survival analysis were available for 184 patients; 25 deaths were observed, and 159 patients (86.4%) were censored. The median follow-up period was 11.5 months (IQR, 2.6–33.1 months; range, 0.13–137.8 months). Detailed clinical data were available for a subset of the cohort, although the completeness of individual variables varied. Available clinical information included Ann Arbor stage, IPI score, LDH, ECOG performance status, extranodal and bone marrow involvement, B-symptom status, treatment regimen, treatment response, and cell-of-origin classification. The number of evaluable patients for each variable is provided in Table 1. Among the 65 patients with available treatment-regimen data, 57 (87.7%) received R-CHOP or an R-CHOP-based regimen, whereas 8 (12.3%) received other systemic treatment regimens. Patients with and without available treatment-response data did not differ significantly with respect to age (median, 63 vs. 65 years; *p* = 0.490), sex (*p* = 0.886), or cell-of-origin classification (*p* = 0.930).

**Figure 1:**
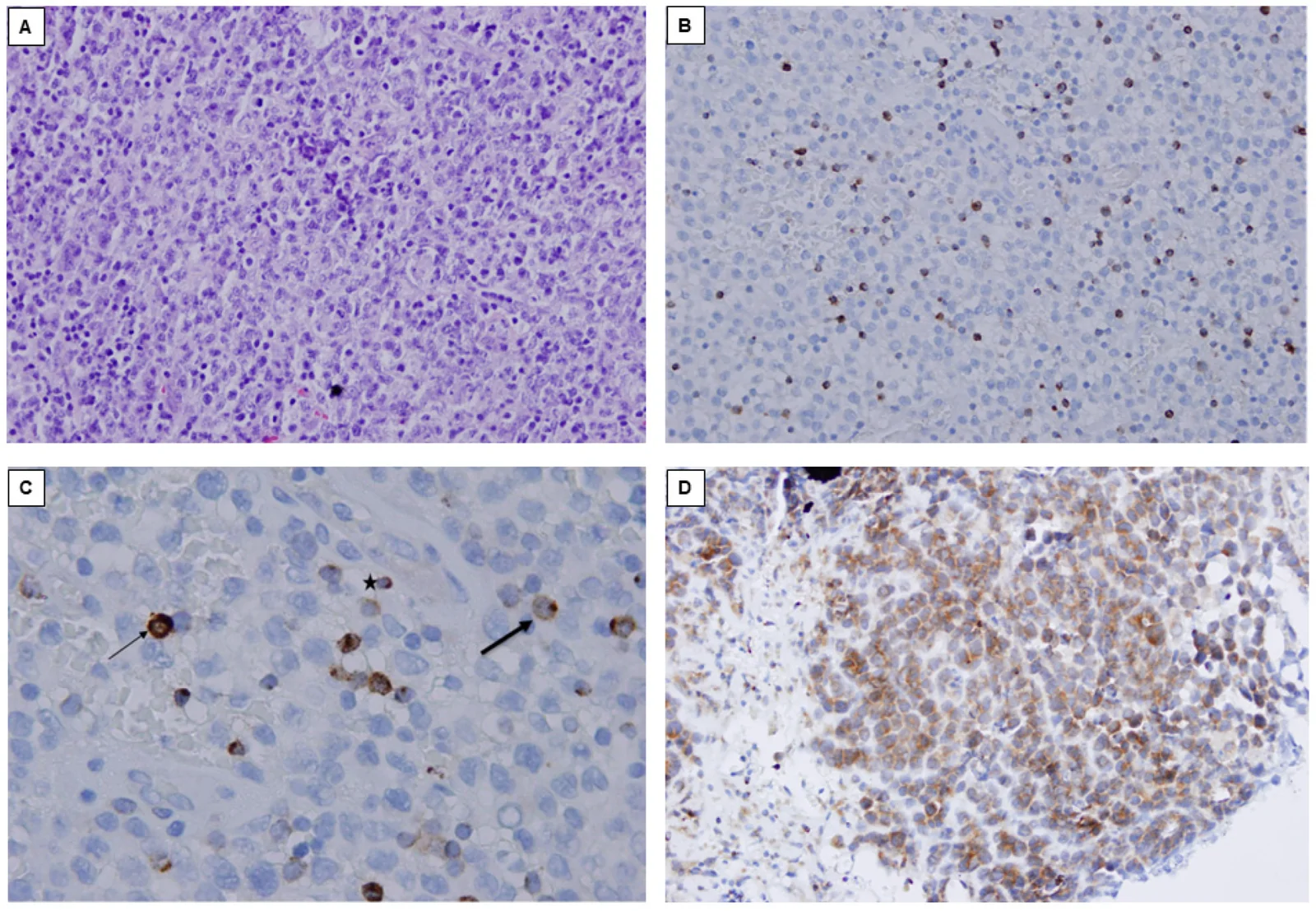
Immunohistochemical expression patterns of LAG-3 in diffuse large B-cell lymphoma. (**A**) Representative hematoxylin and eosin-stained section of DLBCL (×200). (**B**) LAG-3 immunohistochemistry of the corresponding tumor showing LAG-3 expression (×200). (**C**) LAG-3 expression in neoplastic cells demonstrating Golgi-pattern staining (thick arrow) and membranous/cytoplasmic staining (thin arrow); a LAG-3-positive tumor-infiltrating lymphocyte showing a Golgi-pattern staining is indicated by an asterisk (×1000). (**D**) Strong and extensive LAG-3 expression in neoplastic DLBCL cells.

LAG-3 expression of varying intensity was observed in neoplastic cells in 177 of 210 cases (84.3%), whereas 33 cases (15.7%) showed no tumor-cell staining. Weak staining was the predominant pattern (72.9%), followed by moderate (9.5%) and strong (1.9%) staining. Using the predefined 10% cut-off, 82 cases (39.0%) were classified as tumor-cell LAG-3 positive. Representative immunohistochemical LAG-3 staining patterns are shown in Figure 1.

The number of LAG-3-positive TILs varied considerably among cases, with a median of 5 TILs/HPF. The majority of cases showed relatively low numbers of LAG-3-positive TILs.

Male patients had a significantly higher percentage of LAG-3-positive tumor cells than female patients (*p* = 0.039, Mann–Whitney U test). No significant association was observed between sex and the number of LAG-3-positive TILs or tumor-cell staining intensity (Table 2).

A significant association was observed between the number of LAG-3-positive TILs and tumor-cell LAG-3 staining intensity. Among cases with ≤5 LAG-3-positive TILs/HPF, 7 of 109 showed moderate-to-strong tumor-cell staining, compared with 17 of 101 cases with >5 LAG-3-positive TILs/HPF. Thus, moderate-to-strong tumor-cell staining was significantly more frequent in cases with >5 LAG-3-positive TILs/HPF (*p* = 0.018, Pearson’s chi-square test) (Table 3).

No significant associations were observed between treatment response and LAG-3-positive TIL count (*p* = 0.128), tumor-cell LAG-3 percentage (*p* = 0.995), or tumor-cell staining intensity (*p* = 0.994). Similarly, none of these LAG-3 parameters was significantly associated with vital status (*p* = 0.268, *p* = 0.315, and *p* = 0.676, respectively).

**Figure 2:**
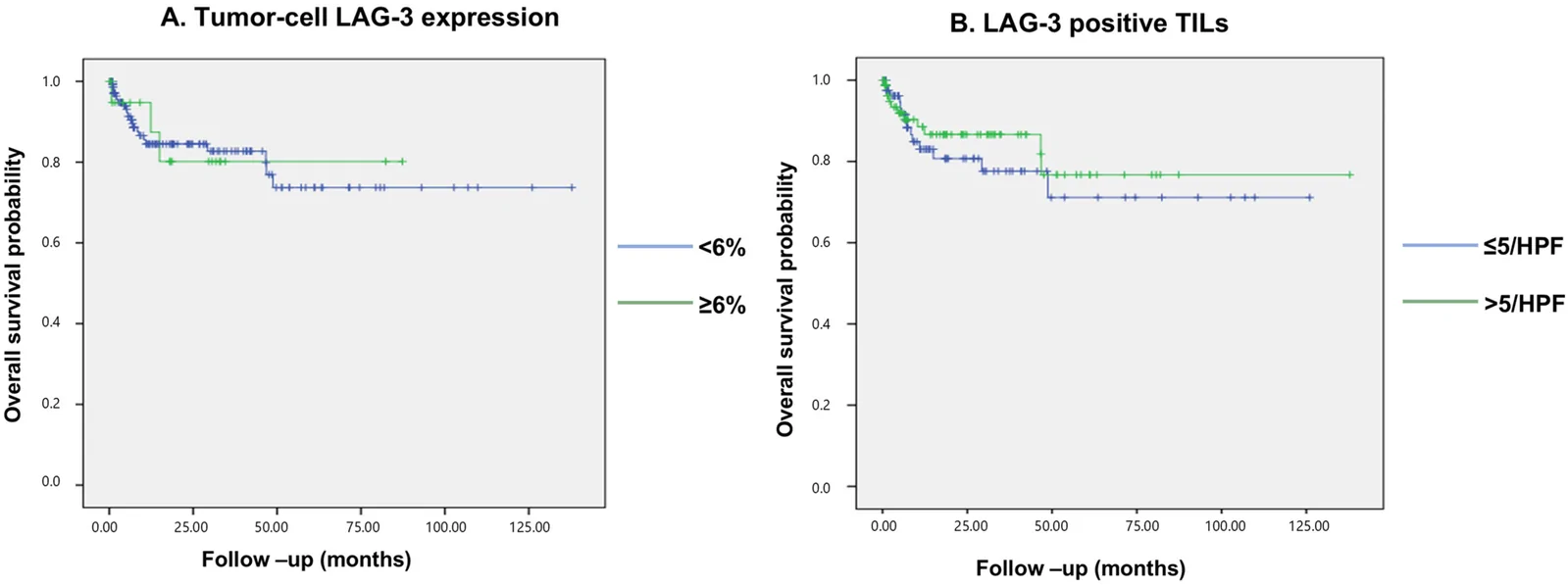
Kaplan–Meier analysis of overall survival according to LAG-3 expression in diffuse large B-cell lymphoma. (**A**) Overall survival according to tumor-cell LAG-3 expression (≤6% vs. >6%; log-rank *p* = 0.310). (**B**) Overall survival according to the number of LAG-3-positive tumor-infiltrating lymphocytes (≤5 vs. >5/HPF; log-rank *p* = 0.493). Tick marks indicate censored observations.

Kaplan–Meier analysis demonstrated no significant difference in overall survival between cases with tumor-cell LAG-3 expression ≤6% and >6% (log-rank *p* = 0.310) (Figure 2A). Similarly, overall survival did not differ significantly between cases with ≤5 and >5 LAG-3-positive TILs/HPF (log-rank *p* = 0.493) (Figure 2B). Analysis according to the presence or absence of tumor-cell staining intensity also showed no significant survival difference (log-rank *p* = 0.626).

PFS data were available for 66 patients, of whom 28 experienced an event. No significant differences in PFS were observed according to LAG-3-positive TIL count (≤5 vs. >5 TILs/HPF; log-rank *p* = 0.677), tumor-cell LAG-3 percentage (≤6% vs. >6%; *p* = 0.993), or tumor-cell staining intensity (0–1+ vs. 2–3+; *p* = 0.637).

In univariate Cox proportional hazards regression analysis, none of the evaluated LAG-3 parameters was significantly associated with overall survival. LAG-3-positive TIL count showed an HR of 0.944 (95% CI, 0.861–1.035; *p* = 0.218). Tumor-cell LAG-3 percentage showed an HR of 1.002 (95% CI, 0.982–1.022; *p* = 0.856), whereas tumor-cell staining intensity showed an HR of 0.847 (95% CI, 0.427–1.683; *p* = 0.636) (Table 4).

## 4. Discussion

In the present study, we evaluated the immunohistochemical expression of LAG-3 in both neoplastic cells and TILs in a cohort of 210 patients with DLBCL. LAG-3 expression was detected in both cellular compartments, with tumor-cell positivity in 39% of cases using a 10% cut-off. The main findings were a significantly higher percentage of LAG-3-positive tumor cells in male patients and an association between increased numbers of LAG-3-positive TILs and stronger LAG-3 expression in neoplastic cells. In contrast, LAG-3 expression was not significantly associated with treatment response or overall survival.

The median number of LAG-3-positive TILs in our cohort was 5 per HPF, which was considerably lower than that reported by Chen et al., who observed a median of 46.1 LAG-3-positive TILs in DLBCL [10]. Differences in antibody characteristics, staining protocols, microscopic field size, and methods used for the selection and quantification of LAG-3-positive lymphocytes may contribute to this discrepancy. Considerable methodological heterogeneity in the assessment of immune checkpoint expression across DLBCL studies emphasizes the importance of standardized approaches when comparing quantitative LAG-3 expression between cohorts [9,10].

An important finding of our study was the demonstration of LAG-3 expression in neoplastic DLBCL cells. Although LAG-3 has traditionally been regarded predominantly as an immune-cell-associated checkpoint molecule [8,13], expression in malignant B cells has also been demonstrated in DLBCL [9,10]. Subsequent studies have further supported LAG-3 expression within the neoplastic compartment of DLBCL [14]. In our cohort, 39% of cases were classified as tumor-cell LAG-3 positive using a 10% cut-off. Differences in the reported frequency of tumor-cell positivity among studies may reflect differences in antibody clones, staining platforms, scoring methods, cellular compartment definitions, and cut-off values. Nevertheless, the expression of LAG-3 in both neoplastic cells and the surrounding immune microenvironment highlights the biological complexity of LAG-3 in DLBCL.

We observed a significantly higher percentage of LAG-3-positive tumor cells in male patients (*p* = 0.039). As the biological basis and reproducibility of this association are currently uncertain, this finding should be considered exploratory and requires confirmation in independent cohorts.

We also found that increased numbers of LAG-3-positive TILs were associated with stronger LAG-3 staining in neoplastic cells. Moderate-to-strong tumor-cell staining was more frequent in cases with >5 LAG-3-positive TILs/HPF than in those with ≤5 positive TILs/HPF (*p* = 0.018). This finding represents an observed association between LAG-3 expression in the neoplastic and immune-cell compartments. Immunohistochemical assessment alone cannot establish biological directionality or causation, and functional studies would be required to clarify the biological significance of this association.

Despite the presence of LAG-3 expression in both tumor cells and TILs, we found no significant association between LAG-3 expression and overall survival. Kaplan–Meier analyses showed no significant differences in overall survival according to tumor-cell LAG-3 expression or LAG-3-positive TIL count. Similarly, univariate Cox proportional hazards regression demonstrated no significant association between overall survival and LAG-3-positive TIL count (HR = 0.944, 95% CI: 0.861–1.035; *p* = 0.218), tumor-cell LAG-3 percentage (HR = 1.002, 95% CI: 0.982–1.022; *p* = 0.856), or tumor-cell staining intensity (HR = 0.847, 95% CI: 0.427–1.683; *p* = 0.636).

Previous studies have reported heterogeneous associations between LAG-3 expression and clinical outcome in DLBCL [9,11,14]. Variable prognostic associations have also been reported in other malignancies, with LAG-3-positive TILs associated with poorer survival in soft tissue sarcoma and favorable clinical outcomes in breast cancer [15,16]. In extranodal NK/T-cell lymphoma, LAG-3 expression has also been demonstrated immunohistochemically, although its clinicopathological and prognostic associations were limited [17]. More recently, increased LAG-3 expression on peripheral blood CD8+ T cells has been associated with poorer progression-free and overall survival in patients with DLBCL [18]. However, this approach evaluates a different cellular compartment from tissue-based immunohistochemistry. Differences in the cellular compartment assessed, antibody and scoring methodology, cut-off values, patient populations, and clinical endpoints may therefore contribute to the heterogeneous prognostic findings across studies.

The absence of a significant survival association in our cohort should also be interpreted in the context of the limited number of observed events. Only 25 deaths occurred during the study period, which may have limited the statistical power to detect modest prognostic effects. Thus, our findings do not exclude a potential prognostic role for LAG-3 but indicate that LAG-3 expression assessed by immunohistochemistry alone was not a significant predictor of overall survival in this cohort. The relatively short median follow-up further limits the precision of the survival estimates and precludes definitive conclusions regarding the prognostic significance of LAG-3. Similarly, exploratory PFS analysis in the subset with available follow-up data showed no significant association between PFS and LAG-3-positive TIL count, tumor-cell LAG-3 percentage, or tumor-cell staining intensity.

From a clinical perspective, our findings do not currently support the use of LAG-3 immunohistochemistry as an independent prognostic biomarker in routine DLBCL practice. However, the expression of LAG-3 in both neoplastic cells and TILs, and their intercompartmental association, provide a rationale for further investigation of LAG-3 as a potential therapeutic target in larger, standardized cohorts.

The main contribution of this study is the simultaneous characterization of LAG-3 expression in both neoplastic cells and TILs within a relatively large DLBCL cohort, allowing direct comparison between these two cellular compartments. In addition, we identified a significant association between increased LAG-3-positive TIL density and stronger tumor-cell LAG-3 staining intensity. These findings support the value of evaluating LAG-3 expression in both neoplastic and immune-cell compartments and highlight the need for standardized assessment methods in future studies.

Several limitations of this study should be acknowledged. First, its retrospective and single-center design may have introduced selection bias. Second, complete clinical information was not available for all patients, particularly for Ann Arbor stage, IPI score, and treatment response, limiting comprehensive clinicopathological and multivariable survival analyses. Third, the relatively small number of death events reduced the statistical power of survival analyses. Finally, standardized criteria for the immunohistochemical assessment of LAG-3 in DLBCL have not been established, and differences in antibody characteristics, staining procedures, field selection, and scoring thresholds may affect comparisons between studies [9,10,11]. Nevertheless, the relatively large pathological cohort and the simultaneous assessment of LAG-3 in both neoplastic cells and TILs represent strengths of the present study and provide additional information regarding the distribution of LAG-3 within the DLBCL tumor microenvironment.

## 5. Conclusions

LAG-3 expression in DLBCL was observed not only in tumor-infiltrating lymphocytes but also in neoplastic B cells, with 39% of cases classified as tumor-cell LAG-3 positive using a 10% cut-off. Tumor-cell LAG-3 expression was higher in male patients, and increased numbers of LAG-3-positive TILs were associated with stronger tumor-cell staining. However, LAG-3 expression was not significantly associated with treatment response or overall survival. These findings warrant confirmation in larger, multicenter cohorts with more complete clinical data and standardized immunohistochemical assessment methods.

## Figures and Tables

**Table 1:** Clinical characteristics of the study cohort.

Characteristic	*n* (%)
**Age, median (IQR)**	62 (51–71)
**Sex**	
Male	102 (48.6)
Female	108 (51.4)
**Cell of origin (*n* = 180)**	
GCB	74 (41.1)
ABC/non-GCB	106 (58.9)
**Ann Arbor stage (*n* = 68)**	
I	6 (8.8)
II	12 (17.6)
III	18 (26.5)
IV	32 (47.1)
**IPI score (*n* = 64)**	
0	1 (1.6)
1	7 (10.9)
2	14 (21.9)
3	25 (39.1)
4	14 (21.9)
5	3 (4.7)
**ECOG performance status (*n* = 62)**	
1	39 (62.9)
2	19 (30.6)
3	4 (6.5)
**B symptoms (*n* = 64)**	
Absent	27 (42.2)
Present	37 (57.8)
**Extranodal involvement (*n* = 67)**	
Absent	37 (55.2)
Present	30 (44.8)
**Bone marrow involvement (*n* = 64)**	
Absent	56 (87.5)
Present	8 (12.5)
**Treatment regimen (*n* = 65)**	
R-CHOP/R-CHOP-based	57 (87.7)
Other	8 (12.3)
**Radiotherapy (*n* = 65)**	
No	54 (83.1)
Yes	11 (16.9)
**Treatment response (*n* = 69)**	
Complete response	48 (69.6)
Partial response	3 (4.3)
No response	18 (26.1)
**Vital status**	
Alive	185 (88.1)
Dead	25 (11.9)
**Follow-up, months**	11.5 (0.13–137.8) †

Percentages were calculated based on the number of patients with available data for each variable. † Data are presented as median (range).

**Table 2:** Distribution of tumor-cell LAG-3 staining intensity according to sex.

Tumor-Cell LAG-3 Staining Intensity	Male, *n* (%)	Female, *n* (%)	*n* (%)
Absent (0)	14 (13.7)	19 (15.7)	33 (15.7)
Weak (1+)	78 (76.5)	75 (72.9)	153 (72.9)
Moderate (2+)	10 (9.8)	10 (9.5)	20 (9.5)
Strong (3+)	0 (0.0)	4 (1.9)	4 (1.9)
Total	102 (100)	108 (100)	210 (100)

**Table 3:** Association between LAG-3-positive TIL count and tumor-cell LAG-3 staining intensity.

LAG-3-Positive TILs	Absent/Weak Staining, *n* (%)	Moderate/Strong Staining, *n* (%)	Total	*p* Value
≤5/HPF	102 (93.6)	7 (6.4)	109	
>5/HPF	84 (83.2)	17 (16.8)	101	**0.018**
Total	186	24	210	

**Abbreviations:** HPF, high-power field; TILs, tumor-infiltrating lymphocytes. *p* value was calculated using Pearson’s chi-square test.

**Table 4:** Univariate Cox proportional hazards regression analysis for overall survival.

Variable	HR	95% CI	*p* Value
LAG-3-positive TIL count	0.944	0.861–1.035	0.218
Tumor-cell LAG-3-percentage	1.002	0.982–1.022	0.856
Tumor-cell LAG-3-intensity	0.847	0.427–1.683	0.636

**Abbreviations:** CI, confidence interval; HR, hazard ratio; TIL, tumor-infiltrating lymphocyte.

## Data Availability

The data supporting the findings of this study are available from the corresponding author upon reasonable request.

## Abbreviations

The following abbreviations are used in this manuscript:

DLBCL	Diffuse large B-cell lymphoma
LAG-3	Lymphocyte activation gene-3
TILs	Tumor-infiltrating lymphocytes
TME	Tumor microenvironment
HPF	High-power field
FFPE	Formalin-fixed paraffin-embedded
IPI	International Prognostic Index
HR	Hazard ratio
CI	Confidence interval
